# Supplementary material for: Are Reproductive Traits Related to Pollen Limitation in Plants? A Case Study from a Central European Meadow
Source: Plants (Basel). 2020 May 19;9(5):640. doi: 10.3390/plants9050640 (PMC7285000; doi:10.3390/plants9050640)
Supplement: Supplementary file 1 [file plants-09-00640-s001.pdf]

Table S1. Correlation matrix of selected traits.

	**Specialization**	**Clonality**	**Dichogamy**	**Sugar Content**	**No. of Open Flowers**	**Self-Compatibility**	**Autonomous Selfing**
Specialization							
Clonality	−0.29						
Dichogamy	**−0.47**	**0.43**					
Sugar content	**0.68**	−0.36	−0.40				
No. of open flowers	0.01	0.01	−0.30	−0.14			
Self-compatibility	0.30	−0.07	−0.27	0.31	−0.04		
Autonomous selfing	−0.08	−0.21	−0.02	−0.09	−0.12	**0.50**	
